# Earlier endovascular therapy within 24 h of ischemic stroke is associated with attenuated systemic inflammation and better neurological recovery

**DOI:** 10.3389/fneur.2026.1759402

**Published:** 2026-03-30

**Authors:** Yan Yan, Yu Qiao, Tengfei Guo, Liyun Wang, Lin Wang

**Affiliations:** 1 Department of Neurology VI, Xingtai People’s Hospital, Xingtai, Hebei, China; 2 Department of Neurology IV, Xingtai People’s Hospital, Xingtai, Hebei, China; 3 Department of Neurology I, Longyao County Hospital, Longyao, Hebei, China

**Keywords:** acute ischemic stroke, endovascular therapy, large-vessel occlusion, neuroinflammation, treatment timing

## Abstract

**Background:**

The impact of endovascular therapy (EVT) timing within the 24-h window on tissue injury and post-ischemic inflammation in large-vessel occlusion stroke remains uncertain.

**Methods:**

In a single-center prospective cohort, 216 anterior circulation large-vessel occlusion patients treated with EVT ≤ 24 h were stratified by onset-to-groin time: 0–6 h (*n* = 74), 6–12 h (*n* = 72), and 12–24 h (*n* = 70). Serial IL-6, TNF-*α*, IL-1β, MMP-9, C-reactive protein, and neutrophil-to-lymphocyte ratio were measured to 72 h; reperfusion quality, final infarct volume, infarct progression, NIHSS change, and 90-day modified Rankin Scale (mRS) were compared.

**Results:**

Baseline demographics, risk factors, stroke severity, and inflammatory profiles were similar across groups. Earlier EVT yielded higher mTICI 2b–3 rates and more first-pass effect, with fewer device passes and shorter procedures. Final infarct volume increased stepwise with delay (38.7, 51.3, and 64.5 mL in the 0–6 h, 6–12 h, and 12–24 h groups), paralleled by greater infarct progression. NIHSS improvement at 24 h and day 7 was greatest in the 0–6 h group, and functional independence at 90 days declined with later treatment (mRS 0–2: 64.9% *vs* 50.0% *vs* 35.7%). Symptomatic intracranial hemorrhage, procedural complications, and in-hospital mortality were low and comparable. Patients treated within 0–6 h showed blunted peaks and faster resolution of IL-6, MMP-9, C-reactive protein, and neutrophil-to-lymphocyte ratio, consistent with a more favorable neuroinflammatory trajectory. These associations remained consistent in adjusted and sensitivity analyses.

**Conclusion:**

Within 24 h, earlier EVT was associated with more favorable reperfusion metrics, smaller infarct burden, lower circulating inflammatory biomarkers, and better 90-day functional outcome, without an apparent increase in major safety events. Given the observational design, these findings should be interpreted as associations rather than causal effects.

## Introduction

Acute ischemic stroke (AIS) remains a principal cause of long-term neurological disability and mortality worldwide, accounting for nearly 70–80% of all stroke cases and placing immense societal and economic burdens on health systems (1). The pathogenesis of AIS is initiated by a sudden occlusion of a cerebral artery, leading to abrupt interruption of regional cerebral blood flow and rapid bioenergetic collapse in neuronal, glial, and endothelial populations (2). Within minutes of vascular blockage, the ischemic core develops irreversible infarction, while the surrounding penumbra—a metabolically compromised but potentially salvageable tissue zone—exhibits active cellular survival pathways and remains sensitive to reperfusion (3). Historically, stroke management has predominantly focused on recanalization, either by intravenous thrombolysis or mechanical thrombectomy, to restore blood flow and minimize infarct extension (4). Although pivotal trials have demonstrated the efficacy of these reperfusion strategies, unfavorable neurological outcomes remain common, even when vascular patency is successfully achieved (5). This therapeutic paradox underscores an urgent need to reconsider the biological mechanisms that mediate post-ischemic brain injury beyond occlusive vascular obstruction and suggests that the timing of endovascular therapy (EVT) may play a decisive role in modulating the immunopathological cascade that unfolds after stroke.

Increasing evidence indicates that ischemic brain injury is fundamentally a neuroinflammatory disorder in addition to a vascular occlusion phenomenon (6). Within the first hours following cerebral ischemia, resident microglia rapidly transform into an activated phenotype, producing cytokines including interleukin-1β (IL-1β), tumor necrosis factor-*α* (TNF-α), and interleukin-6 (IL-6), which amplify blood–brain barrier (BBB) dysfunction and propagate a secondary wave of tissue damage (7). Simultaneously, endothelial upregulation of adhesion molecules such as ICAM-1 and VCAM-1 enables infiltration of circulating neutrophils and monocytes, which further exacerbate oxidative stress, protease activity, and white matter injury (8). The inflammatory response, although originally intended to promote debris clearance and tissue repair, becomes maladaptive when dysregulated, leading to delayed infarct expansion, peri-lesional edema, and neuronal apoptosis (9). These immunologically mediated events exhibit a distinct temporal progression; they begin before reperfusion therapies are typically implemented and remain active well into the subacute and chronic phases (10). Consequently, an intervention that restores perfusion earlier—within the window of microglial activation and before extensive recruitment of peripheral leukocytes—may exert a protective effect by attenuating the neuroinflammatory cascade at its mechanistic point of origin. Understanding this temporal biology is essential to explain why early endovascular treatment may yield profound benefits beyond simple vessel recanalization.

Although endovascular thrombectomy has become standard treatment for AIS with large vessel occlusions (LVO), current practice largely reflects logistical rather than mechanistic considerations (11). Randomized trials have consistently emphasized the 6–24 h therapeutic window, guided by perfusion imaging and tissue viability paradigms such as DAWN and DEFUSE-3 (12). These studies established the principle that salvageable penumbra may persist despite delayed reperfusion, encouraging treatment up to 24 h after onset (13). However, the dominant biological focus in these trials was penumbral persistence, not the underlying neuroinflammatory kinetics. The operational assumption—“time is brain”—remains correct but incomplete; it considers neuronal survival but neglects immunological escalation. Neuroinflammatory progression is nonlinear and accelerates within the first 24 h, with peak neutrophil infiltration occurring as early as 12–18 h, coinciding with maximal BBB permeability and gliotic activation (14). Observational cohorts have reported that earlier EVT correlates with reduced infarct volume, lower rates of symptomatic intracranial hemorrhage, and improved functional outcomes at 90 days (15). Yet, a mechanistic explanation for these observations has not been systematically established, and few clinical investigations have explicitly interrogated how rapid reperfusion may influence neuroimmune modulation. The absence of mechanistic integration has left a clinically relevant knowledge gap: whether EVT administered within 24 h confers benefit not merely through reperfusion but by attenuating the immunopathological cascade responsible for delayed neurological deterioration.

The cerebral immune response following reperfusion is profoundly context-dependent (16). Recanalization achieved after prolonged ischemia may paradoxically worsen tissue injury through reperfusion–inflammation, involving oxygen-derived radicals, mitochondrial failure, and sterile immune activation mediated by pattern recognition receptors such as TLR4 and NLRP3 (17). Late EVT often occurs after extensive leukocyte infiltration and endothelial damage have already been established, increasing susceptibility to hemorrhagic transformation and microvascular collapse (18). In contrast, EVT performed earlier—within the first 6–24 h—may restore perfusion before irreversible microvascular injury and inflammatory amplification have occurred, thereby minimizing reperfusion–immune synergy (19). Experimental rodent models have demonstrated that early reperfusion preserves BBB integrity, reduces microglial overactivation, and limits inflammasome activation (20). Similarly, emerging clinical biomarker studies indicate that early intervention is associated with lower serum levels of IL-6, decreased neutrophil-to-lymphocyte ratios, and reduced plasma matrix metalloproteinase-9 activity (21). These findings strongly suggest that the therapeutic value of EVT transcends mechanical thrombus removal: it may function as a biologically timed immunomodulatory intervention that redefines the trajectory of post-stroke recovery. Yet, this perspective has not been adequately incorporated into clinical discourse, and prospective trials rarely stratify outcomes based on immune signatures or intervention timing relative to inflammatory dynamics.

Understanding the relationship between EVT timing and neuroinflammation is essential for redefining stroke treatment paradigms. Delayed neurological deterioration—even in patients with successful reperfusion—remains a significant clinical challenge (22). Infarct growth, delayed edema formation, and persistent cognitive impairment frequently emerge in the days and weeks following stroke, despite technically optimal endovascular procedures (23). These sequelae are increasingly understood to be mediated by inflammatory persistence within the peri-infarct zone, involving sustained microglial M1 polarization, T-cell recruitment, oligodendrocyte damage, and inhibitory scarring (24). The clinical significance of these phenomena is profound: patients who show early radiological reperfusion often fail to achieve long-term functional independence (25). Thus, a treatment framework that accounts for temporally controlled inflammatory modulation may offer transformative therapeutic benefit. Early EVT may constitute such a framework by interrupting the immunological cascade and shifting the trajectory of neural repair toward a neuroprotective, pro-regenerative phenotype (26). In this context, neuroplasticity, angiogenesis, and synaptic recovery—central pillars of neurological rehabilitation—are more likely to proceed in the absence of persistent inflammatory insult (27).

The present study was designed to test whether, among patients with anterior-circulation large-vessel occlusion treated with EVT within 24 h, earlier onset-to-groin puncture time is associated with improved neurological recovery. Our primary hypothesis was that earlier EVT (0–6 h vs. 6–12 h vs. 12–24 h) is associated with a higher likelihood of functional independence at 90 days (mRS 0–2), alongside smaller infarct burden and more favorable reperfusion performance. As secondary, biologically oriented hypotheses, we anticipated that earlier EVT would be associated with attenuated peaks and faster resolution of a prespecified panel of circulating inflammatory markers (IL-6, TNF-*α*, IL-1β, MMP-9, CRP, and NLR), and that these trajectories would parallel imaging-based tissue injury. Because this prospective cohort quantified peripheral blood biomarkers rather than direct CNS immune activity or BBB integrity, any mechanistic interpretation is necessarily exploratory; therefore, we interpret biomarker differences as clinical correlates that are consistent with, but do not by themselves establish, reduced neuroinflammatory activation.

## Methods

### Ethics statement

All procedures involving human participants were conducted in accordance with the ethical standards of the Institutional Human Research Ethics Committee and the Declaration of Helsinki. Written informed consent was obtained from all patients or their legally authorized representatives prior to enrollment. The study protocol was approved by the Medical Ethics Committee of Xingtai People’s Hospital [Approval Letter No. 2024(031)]. Patient identification data were fully anonymized and stored in a secure database accessible only to study investigators.

### Study design and sample size determination

**Figure 1:**
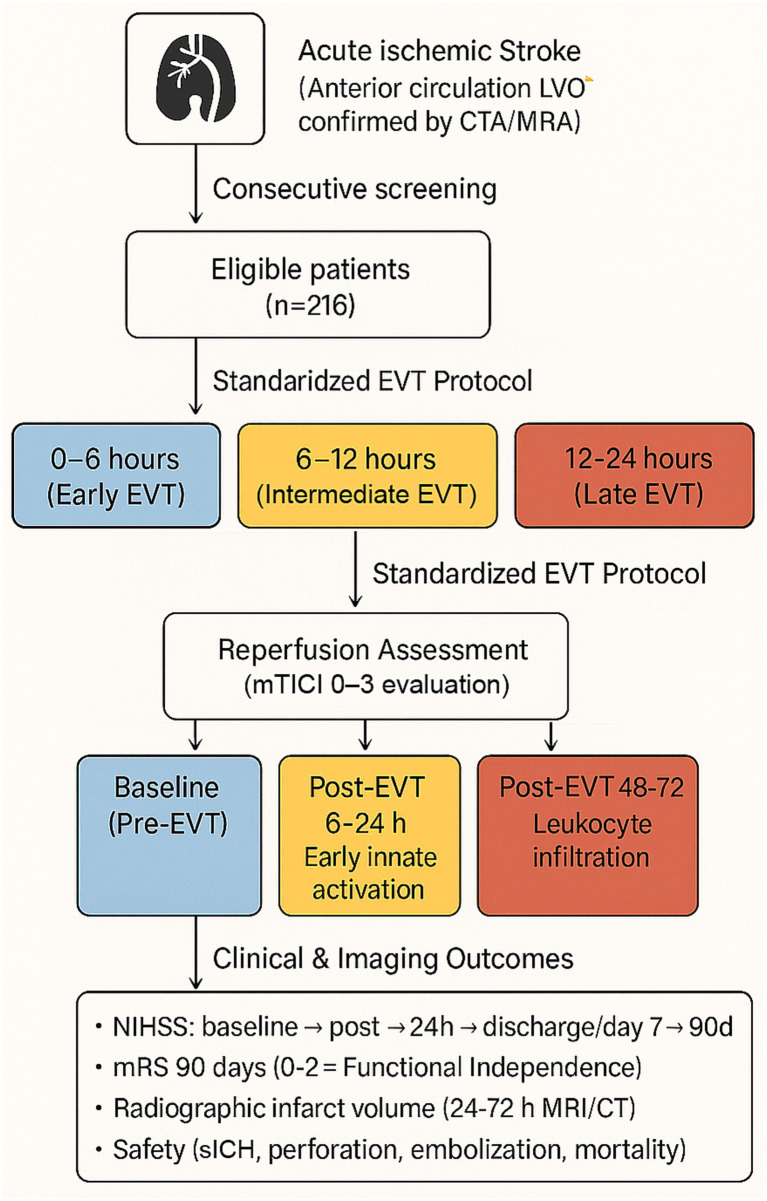
Overall study schema of enrollment, reperfusion timing stratification, neuroinflammatory sampling, and clinical outcomes. **(Step 1)** Consecutive enrollment and eligibility. Patients presenting with suspected acute ischemic stroke underwent vascular imaging to confirm anterior circulation large-vessel occlusion (intracranial ICA or M1/M2 MCA). Individuals meeting predefined inclusion and exclusion criteria were enrolled, yielding a final cohort of 216 eligible patients. **(Step 2)** Endovascular therapy timing groups. Participants were stratified according to the interval from symptom onset (or last known well) to initiation of EVT into three predefined categories: 0–6 h (early EVT), 6–12 h (intermediate EVT), and 12–24 h (late EVT). **(Step 3)** Neuroinflammatory biomarker sampling timeline. Peripheral venous blood was collected at three standardized windows to capture the temporal dynamics of post-stroke innate and peripheral immune activation: baseline prior to EVT, 6–24 h post-EVT (early innate activation), and 48–72 h post-EVT (leukocyte recruitment and delayed inflammatory amplification). **(Step 4)** Clinical and imaging outcomes. Neurological status was assessed using the National Institutes of Health Stroke Scale (NIHSS) at serial timepoints, and functional status was evaluated at 90 days using the modified Rankin Scale (mRS), with independence defined as mRS 0–2. Secondary outcomes included radiographic infarct volume on follow-up CT/MRI and safety endpoints such as symptomatic intracerebral hemorrhage, vessel perforation, distal embolization, and in-hospital mortality.

This single-center prospective observational study was conducted at a tertiary stroke referral institution equipped with a dedicated comprehensive endovascular unit. Figure 1 provides an overview of the study framework, illustrating the sequence of patient enrollment, categorization according to EVT initiation time, predefined neuroinflammatory sampling intervals, and the evaluation of clinical outcomes. Over a continuous 3-year enrollment period, all consecutively presenting patients with acute ischemic stroke secondary to anterior circulation large-vessel occlusion were systematically screened for eligibility. An *a priori* sample size calculation was performed to ensure adequate power to detect a clinically meaningful improvement in functional outcomes associated with early EVT. The calculation was based on an anticipated absolute 20% increase in the rate of functional independence (modified Rankin Scale [mRS] 0–2 at 90 days) among patients treated within the early intervention window. Assuming a two-sided *α* of 0.05, statistical power of 80%, and a baseline independence rate of 35%, a minimum of 186 participants was required. To account for an estimated 10% loss to follow-up or incomplete outcome acquisition, the target enrollment was set at ≥205 patients. Ultimately, 216 patients were included in the final analytic cohort, exceeding the calculated threshold and providing >80% power to detect an odds ratio of at least 1.8 for functional independence. All EVT procedures, neuroimaging assessments, and post-procedural evaluations were performed in accordance with standardized institutional protocols and contemporary evidence-based clinical practice guidelines.

### Definition and rationale for EVT timing strata

EVT timing was categorized as 0–6 h, 6–12 h, and 12–24 h from symptom onset (or last known well) to groin puncture. The 0–6 h threshold corresponds to the conventional early treatment window in which benefit is consistently greatest with shorter onset-to-reperfusion times. The 6–24 h window aligns with the evidence base supporting thrombectomy in late-presenting patients selected by advanced imaging, as established in the DAWN and DEFUSE 3 trials, and reflected in contemporary guideline recommendations for carefully selected anterior-circulation LVO (28, 29). We further subdivided the late window at 12 h to enhance clinical interpretability and to reflect the evolving temporal biology of post-ischemic injury. Experimental and translational studies indicate that early innate immune activation and endothelial barrier stress emerge within hours after LVO, whereas leukocyte recruitment and secondary inflammatory amplification become more prominent later in the first day (30). Accordingly, the 6–12 h and 12–24 h strata were prespecified to contrast an earlier versus later phase within the extended window while avoiding over-fragmentation and loss of statistical power.

### Patient enrollment and eligibility criteria

Patients presenting to the emergency department with suspected acute ischemic stroke were consecutively screened by a multidisciplinary neurovascular team over the three-year study period, and eligibility was determined based on clinical, radiological, and procedural requirements to ensure suitability for endovascular intervention. Inclusion criteria were: (1) age ≥ 18 years; (2) diagnosis of acute ischemic stroke involving anterior circulation large vessel occlusion confirmed by vascular imaging of the intracranial internal carotid artery or the M1/M2 segments of the middle cerebral artery; (3) initiation of EVT within 24 h of symptom onset, determined by the last-known-well time or a clearly witnessed onset. For unwitnessed-onset presentations, including wake-up strokes, symptom onset was operationally defined using the last-known-well time, that is, the last time the patient was documented or reported to be neurologically intact. For wake-up strokes specifically, the last-known-well time corresponded to the time the patient was last seen well before sleep. The time of symptom discovery was recorded separately but was not used to assign EVT timing strata. To minimize misclassification, onset-related information was triangulated from emergency medical service documentation, contemporaneous medical records, and caregiver or witness reports, and was independently adjudicated by two senior stroke neurologists with discrepancies resolved by consensus; (4) baseline neurological severity with a National Institutes of Health Stroke Scale (NIHSS) score ≥ 6 (31); and (5) absence of intracranial hemorrhage on initial non-contrast computed tomography. Exclusion criteria included: (1) pre-stroke functional dependence defined as a modified Rankin Scale score ≥ 3; (2) history of intracranial hemorrhage, vasculitis, moyamoya disease, or other cerebrovascular malformations deemed contraindications for thrombectomy; (3) severe coagulopathy or ongoing anticoagulation with uncontrolled laboratory parameters that would contraindicate EVT; and (4) terminal illness or systemic conditions expected to limit adequate clinical follow-up. In cases where symptom onset time could not be clearly established, clinical documentation, emergency medical records, and caregiver or witness information were independently reviewed by two senior stroke neurologists, and any disagreements were resolved through consensus to ensure accurate classification of treatment window eligibility.

### Neuroimaging acquisition and analysis

All patients underwent immediate non-contrast cranial computed tomography (CT) upon admission to exclude intracranial hemorrhage and to quantify the extent of early ischemic change using the Alberta Stroke Program Early CT Score (ASPECTS). Vascular imaging with CT angiography or magnetic resonance angiography was subsequently performed to confirm the presence of anterior circulation large vessel occlusion and characterize the anatomical location of the thrombus, including intracranial internal carotid artery segments and M1/M2 divisions of the middle cerebral artery. Collateral circulation was not systematically graded in this cohort. Although baseline CTA/MRA was routinely performed to confirm large-vessel occlusion, standardized collateral scoring (e.g., multiphase CTA–based collateral scales or DSA collateral grades) was not available for all patients under a uniform protocol; therefore, collateral status was not included as a covariate in the primary models. We partially mitigated this limitation by adjusting for baseline ischemic burden (ASPECTS), occlusion location, and perfusion-derived parameters when available. When clinically indicated, perfusion imaging was obtained to evaluate tissue viability and perfusion–diffusion mismatch according to institutional protocols, and automated post-processing software was used to generate quantitative maps of cerebral blood flow, cerebral blood volume, and time-to-maximum. Reperfusion following EVT was graded on final angiographic runs using the modified Thrombolysis in Cerebral Infarction (mTICI) scale, with successful reperfusion defined as mTICI 2b–3. Postprocedural follow-up imaging was performed within 24–72 h to document infarct progression, hemorrhagic transformation, and postoperative complications; infarct volume was calculated via automated segmentation, and in cases of algorithmic failure or imaging artifacts, regions of interest were manually delineated by a fellowship-trained neuroradiologist. To ensure consistency and reduce observer bias, imaging data were reviewed by clinicians blinded to clinical outcomes, and a random sample comprising 20% of the dataset was independently re-evaluated by a second neuroradiologist; interobserver reliability was quantified using intraclass correlation coefficients.

### EVT protocol

All procedures were performed in a dedicated neurointerventional suite by board-certified neuroradiologists with a minimum of 5 years of independent thrombectomy experience, following institutional standardized operating procedures and contemporary international stroke intervention guidelines. To minimize learning-curve variability and operator-related performance bias, more than 90% of the procedures were conducted by two senior interventional neuroradiologists, while the remaining procedures were performed by experienced team members under their direct supervision.

Procedures were preferentially performed under conscious sedation with local anesthesia to minimize treatment delays. General anesthesia with endotracheal intubation was selected when clinically indicated, including depressed consciousness or inability to protect the airway, persistent vomiting/aspiration risk, severe agitation precluding safe immobilization, respiratory failure or hemodynamic instability, or an anticipated prolonged/complex procedure at the discretion of the neurointerventionalist and anesthesiologist. The anesthesia strategy was determined immediately on arrival to the angiography suite through a joint decision by the stroke team and anesthesia staff, with an explicit goal of avoiding workflow delay. Conversion from conscious sedation to general anesthesia was permitted when required for patient safety and was recorded.

Vascular access was preferentially obtained through the common femoral artery under ultrasound guidance, with a radial approach reserved for patients with femoral contraindications or unfavorable iliac anatomy. After stable proximal support was established at the cervical internal carotid artery, intracranial navigation was performed under roadmap guidance using a microcatheter–microwire system to reach the thrombus interface. First-line thrombectomy strategy followed a pre-specified algorithm based on vascular anatomy and clot characteristics: aspiration-first (ADAPT) was preferred for proximal M1 occlusions and straight vessel segments to minimize traction injury; stent-retriever deployment was utilized in carotid terminus occlusions, bifurcation thrombi, or distal M2 disease; and combined approaches were adopted when clot length, preprocedural imaging, or initial attempts suggested reduced likelihood of single-modality success. For aspiration-based strategies, controlled negative pressure was applied at the clot face prior to withdrawal along the vessel axis to limit distal embolization and endothelial shearing. For stent retrievers, full coverage across the thrombus was ensured, with a pre-defined embedding interval of 2–5 min for clot-device integration, followed by en bloc retrieval under simultaneous proximal aspiration. Switching criteria were mandatory rather than discretionary: two unsuccessful aspiration passes prompted conversion to retriever-based extraction, and one failed retriever pass triggered escalation to combined thrombectomy. Angiographic assessments were performed after each pass, and reperfusion was graded using the modified Thrombolysis in Cerebral Infarction (mTICI) scale, with mTICI 2b–3 regarded as successful recanalization. The total number of passes was restricted to a maximum of three unless angiographic response suggested high-yield potential for a fourth attempt, to mitigate procedural endothelial injury and hemorrhagic transformation. Rescue techniques—including balloon angioplasty, carotid stenting, or intra-arterial antithrombotic infusion—were permitted only if vessel integrity was preserved, distal circulation remained viable, and hemorrhagic risk was clinically acceptable. Termination criteria were predefined: absence of further reperfusion improvement after repeated attempts, procedural complications such as vessel perforation or dissection, or emergent hemodynamic instability requiring cessation. All complications—including vasospasm, distal embolization, angiographic contrast extravasation, and symptomatic intracranial hemorrhage—were prospectively recorded and independently adjudicated by two interventional neuroradiologists blinded to clinical outcomes.

### Peri-procedural and post-procedural management

Following vascular access and throughout the thrombectomy procedure, continuous invasive hemodynamic monitoring was performed to maintain optimal cerebral perfusion, with systolic blood pressure controlled within predefined target ranges to prevent intra-procedural hypotension and reperfusion-associated hyperperfusion injury. Patients undergoing conscious sedation received supplemental oxygen with capnography monitoring and immediate escalation to airway protection if respiratory compromise or agitation threatened procedural stability, whereas patients under general anesthesia were ventilated with normocapnic targets to avoid CO_2_-mediated alterations in intracranial blood flow. Immediately after reperfusion, all patients were transferred to a dedicated stroke intensive care unit for at least 24 h, receiving standardized post-EVT management that included vigilant neurological assessment at predetermined intervals, continuous cardiopulmonary monitoring, strict temperature regulation, and active glucose control to limit metabolic contributions to neuroinflammatory injury. Early blood pressure management emphasized avoiding abrupt reductions that might jeopardize penumbral perfusion and preventing hypertensive surges that could precipitate hemorrhagic transformation; antihypertensive adjustments were guided by real-time neurological status and post-procedural imaging. Antithrombotic therapy was individualized based on reperfusion quality, presence of residual stenosis, and procedural risk, with dual antiplatelet therapy initiated after procedural safety was confirmed unless contraindicated by hemorrhagic complications; anticoagulation strategies were deferred or escalated according to post-operative imaging and clinical stability. A follow-up brain CT or MRI was obtained within 24–72 h to document infarct evolution, hemorrhagic transformation, or procedure-related vascular injury, and any adverse events—including vasospasm, large-vessel re-occlusion, contrast extravasation, or symptomatic intracranial hemorrhage—were managed according to established neurocritical protocols. Early rehabilitation evaluation was initiated within 48–72 h when clinically feasible to promote neurological recovery, reduce immobilization-related complications, and support post-acute recovery trajectories, and long-term secondary prevention measures were implemented prior to discharge in alignment with stroke guidelines and individualized vascular risk stratification.

### Neuroinflammatory biomarker collection

Peripheral venous blood samples were collected at three prespecified timepoints to characterize the temporal trajectory of post-stroke neuroimmune responses: immediately before EVT to establish a pre-intervention baseline; at 6–24 h post-procedure to capture the early rise in innate inflammatory mediators; and at 48–72 h to reflect the later phase associated with leukocyte recruitment and delayed immune amplification. All samples were processed within 30 min of collection to preserve analyte stability. Whole blood was centrifuged at 1,500 × g for 10 min at 4 °C, and the resulting plasma was aliquoted into sterile cryovials and stored at −80 °C without repeated freeze–thaw cycles. Quantified biomarkers included core systemic neuroinflammatory mediators (interleukin-6, tumor necrosis factor-*α*, IL-1β, C-reactive protein [CRP], and matrix metalloproteinase-9), as well as peripheral immune indices such as the neutrophil-to-lymphocyte ratio (NLR), collectively reflecting innate immune activation, endothelial injury, and peripheral immune recruitment. These biomarkers were prespecified *a priori* to capture complementary and clinically relevant nodes of the post-ischemic inflammatory cascade. IL-1β, TNF-*α*, and IL-6 were selected as canonical early pro-inflammatory cytokines linked to innate immune activation after large-vessel occlusion, whereas MMP-9 was chosen given its established association with blood–brain barrier disruption and hemorrhagic transformation. CRP and the neutrophil-to-lymphocyte ratio were included as pragmatic indices of systemic inflammatory burden and peripheral leukocyte mobilization that are readily obtainable and have been associated with stroke severity and EVT outcomes. Collectively, this parsimonious panel balances biological coverage with feasibility for standardized serial sampling in acute stroke care.

All measurements were performed using validated enzyme-linked immunoassays or automated immunoassay platforms according to manufacturer instructions. For ELISA-based assays, each sample was measured in duplicate and the mean value was used for analysis; assays were repeated if the duplicate coefficient of variation exceeded 10%. Intra-assay and inter-assay variability, expressed as coefficients of variation, were ≤5% and ≤10%, respectively, per manufacturer specifications. For automated platforms, calibration and internal quality controls were performed according to the manufacturers’ recommendations. To minimize analytical drift, all longitudinal samples from the same patient were analyzed within the same batch, and internal positive and negative controls were included on each plate/run to ensure inter-plate consistency. Hemolyzed, lipemic, or visibly contaminated specimens were excluded *a priori*; when volume permitted, duplicate aliquots from the original sample were reprocessed to avoid data loss. Laboratory personnel performing biomarker quantification were blinded to both clinical outcomes and EVT timing group assignment (0–6 h, 6–12 h, 12–24 h); samples were labeled with coded identifiers without timing-group information.

### Clinical outcomes

Neurological severity was assessed using the National Institutes of Health Stroke Scale (NIHSS) at baseline, immediately post-procedure, at 24 h, at day 7 or discharge, and at 90 days to quantify early and delayed neurological recovery. Functional outcomes were evaluated at 90 days using the modified Rankin Scale (mRS), with functional independence defined *a priori* as mRS 0–2, and shifts across the full ordinal mRS distribution were also analyzed to capture gradations of disability. Safety outcomes included the incidence of symptomatic intracranial hemorrhage, defined according to ECASS-III criteria as any hemorrhage associated with neurological deterioration of ≥4 NIHSS points, as well as angiographically documented vessel injury, contrast extravasation, procedure-related embolization, and in-hospital mortality. Secondary imaging outcomes included final infarct volume and radiographic infarct expansion on follow-up CT or MRI obtained within 24–72 h, with lesion progression interpreted relative to baseline neuroimaging. All clinical assessments were performed by trained stroke neurologists who were blinded to procedural timing and biomarker data, and outcome adjudication discrepancies were resolved by consensus. Patients without in-person follow-up at 90 days were contacted through structured telephone interviews using validated scoring methods, and missing functional data were recorded according to pre-specified definitions to minimize follow-up bias.

### Statistical analysis

The primary analytic objective was to estimate the effect of EVT timing on 90-day functional independence while accounting for baseline stroke severity and reperfusion status. EVT timing was modeled as a categorical exposure (0–6 h, 6–12 h, 12–24 h). The primary endpoint (mRS 0–2 at 90 days) was evaluated using multivariable logistic regression. Covariates were prespecified based on biological plausibility and established determinants of thrombectomy response: age, baseline NIHSS, ASPECTS, occlusion segment (ICA/M1/M2), IV-tPA administration, and reperfusion success (mTICI 2b–3). No univariate screening was performed. To mitigate treatment-selection bias, inverse probability weighting was applied using propensity scores derived from EVT timing predictors (age, NIHSS, ASPECTS, occlusion site, IV-tPA). Balance was assessed using standardized mean differences (SMD < 0.10). Weighted models yielded stabilized population-level estimates. Longitudinal biomarker trajectories were analyzed using linear mixed-effects models with random intercepts per patient. Timepoint (baseline, 6–24 h, 48–72 h) and EVT timing were included as fixed effects. The interaction term (timepoint × EVT timing) tested whether early intervention altered inflammatory dynamics independent of reperfusion. Log-transformation was applied when normality assumptions were violated. An unstructured covariance matrix was used unless Akaike criteria favored a simpler structure. Follow-up ascertainment for the primary outcome was complete, with 90-day mRS available for all included participants; therefore, complete-case analysis for the primary endpoint was equivalent to analysis of the full cohort. For secondary variables, missingness was limited and arose primarily from pragmatic, non-systematic reasons, including unavailable follow-up neuroimaging within the prespecified window, insufficient sample volume, or exclusion of hemolyzed/lipemic specimens. We did not impute missing secondary data. Analyses were performed using all available observations, and denominators are reported in the corresponding results where applicable. Longitudinal biomarker analyses used mixed-effects models, which accommodate unbalanced repeated measures under a missing-at-random assumption. No covariates were introduced *post hoc*. Multiplicity was addressed by hierarchical outcome ordering: (1) mRS 0–2, (2) ordinal mRS distribution (proportional odds), (3) biomarker trajectories. Subgroup analyses included restriction to mTICI 2b–3, stratification by anesthesia modality, and exclusion of unwitnessed-onset strokes. Effect modification by anesthesia modality was assessed by including an EVT timing × anesthesia interaction term in the prespecified adjusted models, with interaction *p* values reported. For ordinal mRS analyses, the proportional-odds (parallel-lines) assumption was formally evaluated (Brant test); no meaningful violation was detected, and results are therefore reported from the proportional-odds model. Given modest between-group differences in baseline ischemic burden (ASPECTS), we performed prespecified sensitivity analyses to more explicitly account for ASPECTS. First, we repeated the primary logistic regression and the ordinal (proportional-odds) models with ASPECTS modeled as (i) a continuous covariate and (ii) a categorical variable (e.g., ≤7 vs. ≥ 8) to assess robustness to functional form assumptions. Second, we repeated the primary outcome analyses after restricting the cohort to patients with ASPECTS ≥6 (and, in an additional analysis, ≥7) at baseline to minimize confounding by extensive early ischemic change. Consistency of effect estimates across these analyses was interpreted as supporting robustness to baseline ASPECTS imbalance. Two-sided *p* < 0.05 denoted significance.

## Results

### Patient population and baseline characteristics

A total of 216 patients with anterior circulation large-vessel occlusion undergoing EVT within 24 h of symptom onset were included in the final analysis and stratified into three onset-to-groin time windows: early (0–6 h, *n* = 74), intermediate (6–12 h, *n* = 72), and late (12–24 h, *n* = 70). The study flow and cohort assembly are summarized in Figure 1.

Baseline demographics and vascular risk factors were broadly comparable across the three groups (Table 1). Mean age (66.1 ± 10.7, 67.9 ± 11.2, and 68.5 ± 10.4 years for early, intermediate, and late EVT, respectively; *p* = 0.366) and the proportion of male patients (60.8, 55.6, and 54.3%; *p* = 0.701) did not differ significantly. The prevalence of hypertension, diabetes mellitus, dyslipidemia, atrial fibrillation, coronary artery disease, and current smoking was also similar among timing strata (all *p* > 0.05). Baseline systolic blood pressure and NIHSS scores were comparable (NIHSS 15.2 ± 4.2 *vs* 16.0 ± 4.4 *vs* 15.6 ± 4.5; *p* = 0.516). In contrast, there were modest but significant differences in baseline ischemic burden and workflow intervals. Patients in the late EVT group had lower ASPECTS (7.8 ± 1.6) than those in the early group (8.6 ± 1.2; *p* < 0.05), indicating more extensive early ischemic change with treatment delay. Onset-to-door time and door-to-groin puncture time increased stepwise across groups (62.2 ± 18.1, 71.4 ± 20.3, and 79.5 ± 23.2 min; and 92.2 ± 24.3, 121.5 ± 27.2, and 168.3 ± 32.1 min, respectively; both *p* < 0.001), reflecting progressively longer prehospital and in-hospital delays in patients treated later. Median (IQR) values showed a consistent stepwise increase (Table 1). Baseline infarct core volume increased modestly with treatment delay (*p* = 0.018), consistent with the ASPECTS differences across groups, while the absolute volume remained small in all cohorts. Retrospective collateral grading of 126 patients showed no significant difference in the distribution of good collateral status among the early (68.2%), intermediate (65.9%), and late (62.1%) EVT groups (*p* = 0.689), indicating no baseline collateral imbalance in the subcohort with available data. Perfusion imaging was performed in 108 patients (49.1% of the cohort), with no significant difference in the acquisition rate across EVT timing groups (*p* = 0.723). The median penumbra volume was 42.5 mL (IQR 28.7–56.3) in the early group, 40.2 mL (IQR 26.5–53.8) in the intermediate group, and 38.7 mL (IQR 25.1–51.2) in the late group (*p* = 0.659), with perfusion-diffusion mismatch present in 89.2, 86.1, and 82.9% of patients, respectively (*p* = 0.581). Baseline inflammatory markers, including CRP and NLR, were broadly similar among the three groups (Table 1).

**Table 1:** Baseline characteristics of patients stratified by endovascular therapy initiation time.

Variable	Early EVT (0–6 h, *n* = 74)	Intermediate EVT (6–12 h, *n* = 72)	Late EVT (12–24 h, *n* = 70)	Statistic	*p* value
Demographics					
Age, years	66.1 ± 10.7	67.9 ± 11.2	68.5 ± 10.4	*F* = 1.013	0.366
Male sex, %	45 (60.8%)	40 (55.6%)	38 (54.3%)	*χ*^2^ = 0.710	0.701
BMI, kg/m^2^	24.9 ± 3.8	25.1 ± 3.9	25.4 ± 3.7	*F* = 0.351	0.795
Vascular risk factors and comorbidities					
Hypertension	48 (64.9%)	46 (63.9%)	45 (64.3%)	*χ*^2^ = 0.015	0.992
Diabetes mellitus	19 (25.7%)	21 (29.2%)	20 (28.6%)	*χ*^2^ = 0.254	0.881
Dyslipidemia	28 (37.8%)	30 (41.7%)	27 (38.6%)	*χ*^2^ = 0.251	0.882
Atrial fibrillation	26 (35.1%)	28 (38.9%)	25 (35.7%)	*χ*^2^ = 0.255	0.880
Coronary artery disease	14 (18.9%)	16 (22.2%)	17 (24.3%)	*χ*^2^ = 0.622	0.733
Current smoking	22 (29.7%)	21 (29.2%)	20 (28.6%)	*χ*^2^ = 0.023	0.988
Stroke severity and baseline imaging					
Baseline SBP, mmHg	147.2 ± 21.3	149.6 ± 22.1	151.7 ± 23.3	*F* = 0.942	0.392
Baseline NIHSS	15.2 ± 4.2	16.0 ± 4.4	15.6 ± 4.5	*F* = 0.664	0.516
ASPECTS	8.6 ± 1.2	8.3 ± 1.4	7.8 ± 1.6	*F* = 5.743	0.004
Baseline infarct core volume, mL	12.3 ± 5.8	14.7 ± 6.5	17.2 ± 7.1	*F* = 4.125	0.018
Occlusion anatomy				*χ*^2^ = 1.817	0.769
LVO: ICA	18 (24.3%)	22 (30.6%)	24 (34.3%)		
LVO: M1	41 (55.4%)	37 (51.4%)	33 (47.1%)		
LVO: M2	15 (20.3%)	13 (18.1%)	13 (18.6%)		
Acute workflow intervals					
IV-tPA given	32 (43.2%)	29 (40.3%)	26 (37.1%)	*χ*^2^ = 0.557	0.757
Onset to door, mean±SD + median [IQR], min	62.2 ± 18.1; 60 (49–74)	71.4 ± 20.3; 71 (58–85)	79.5 ± 23.2; 80 (64–95)	*F* = 19.821	<0.001
First-hospital arrival-to-groin puncture time, mean±SD + median [IQR], min	92.2 ± 24.3; 90 (76–109)	121.5 ± 27.2; 118 (103–140)	168.3 ± 32.1; 162 (147–190)	*F* = 120.137	<0.001
Baseline laboratory					
Serum glucose, mmol/L	6.81 ± 1.64	7.04 ± 1.83	7.28 ± 1.99	*F* = 1.532	0.224
CRP baseline, mg/L	5.2 ± 2.3	5.4 ± 2.6	5.7 ± 2.9	*F* = 0.486	0.619
NLR baseline	4.6 ± 1.9	4.8 ± 2.1	5.1 ± 2.3	*F* = 1.021	0.361

Baseline demographic, vascular risk, stroke severity, imaging variables, and workflow intervals are summarized across EVT timing groups. Continuous variables are presented as mean ± SD; for workflow time intervals, median (interquartile range) is additionally provided. Categorical variables are presented as n (%). EVT, endovascular therapy; BMI, body mass index; SBP, systolic blood pressure; NIHSS, National Institutes of Health Stroke Scale; ASPECTS, Alberta Stroke Program Early CT Score; LVO, large-vessel occlusion; ICA, intracranial internal carotid artery; M1/M2, M1/M2 segments of the middle cerebral artery; IV-tPA, intravenous tissue plasminogen activator; CRP, C-reactive protein; NLR, neutrophil-to-lymphocyte ratio.

### Reperfusion performance and procedural characteristics

**Figure 2:**
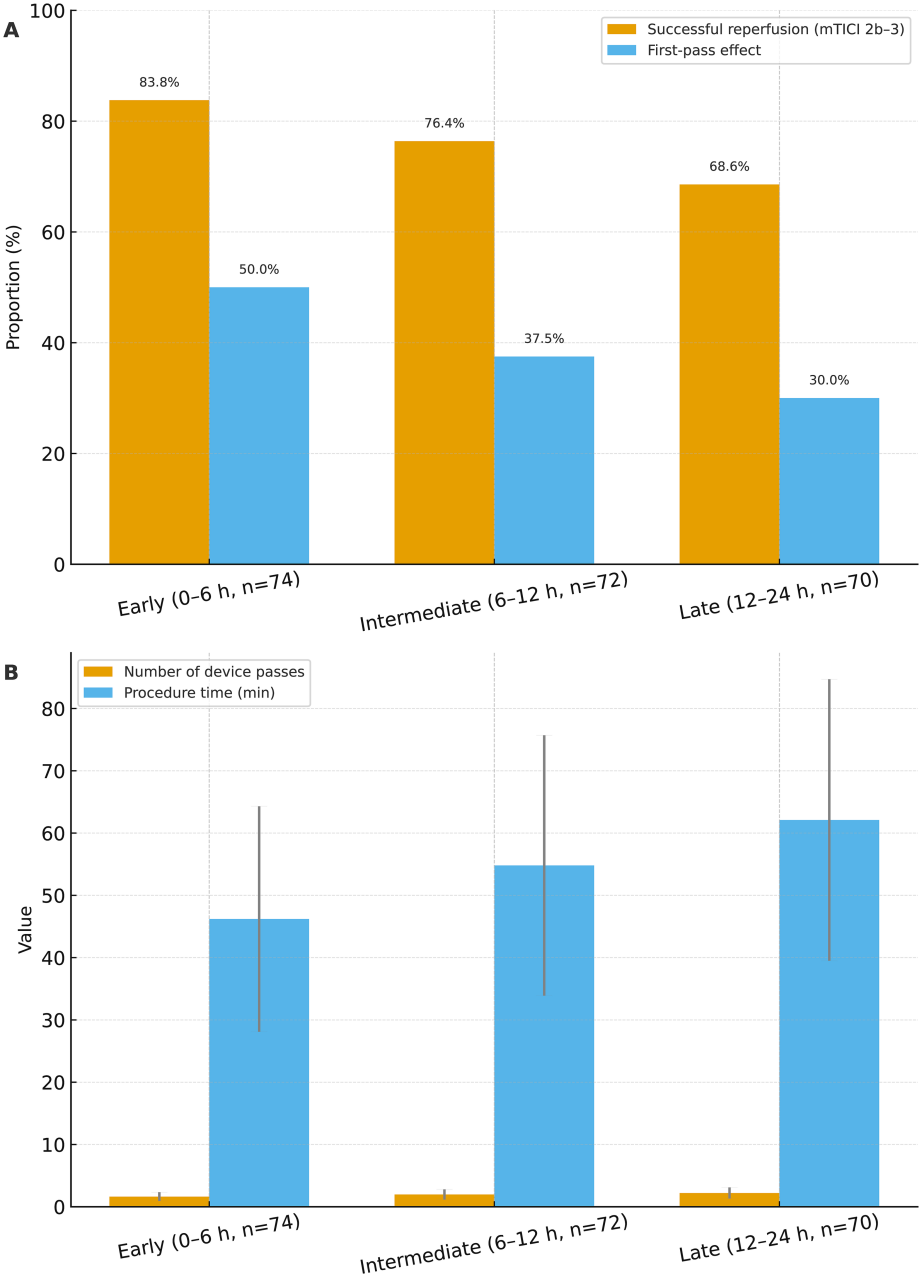
Reperfusion performance and procedural metrics according to timing of endovascular therapy. **(A)** Proportion of patients achieving successful angiographic reperfusion (modified Thrombolysis in Cerebral Infarction [mTICI] grade 2b–3) and first-pass effect in the early (0–6 h, *n* = 74), intermediate (6–12 h, *n* = 72), and late (12–24 h, *n* = 70) endovascular therapy (EVT) groups. Values are expressed as percentages, with exact rates shown above each bar. **(B)** Number of thrombectomy device passes and total procedure time in the three EVT timing groups. Bars represent mean values and error bars represent standard deviation. Group comparisons for categorical and continuous variables were performed using χ^2^ tests and one-way analysis of variance, respectively.

**Figure 3:**
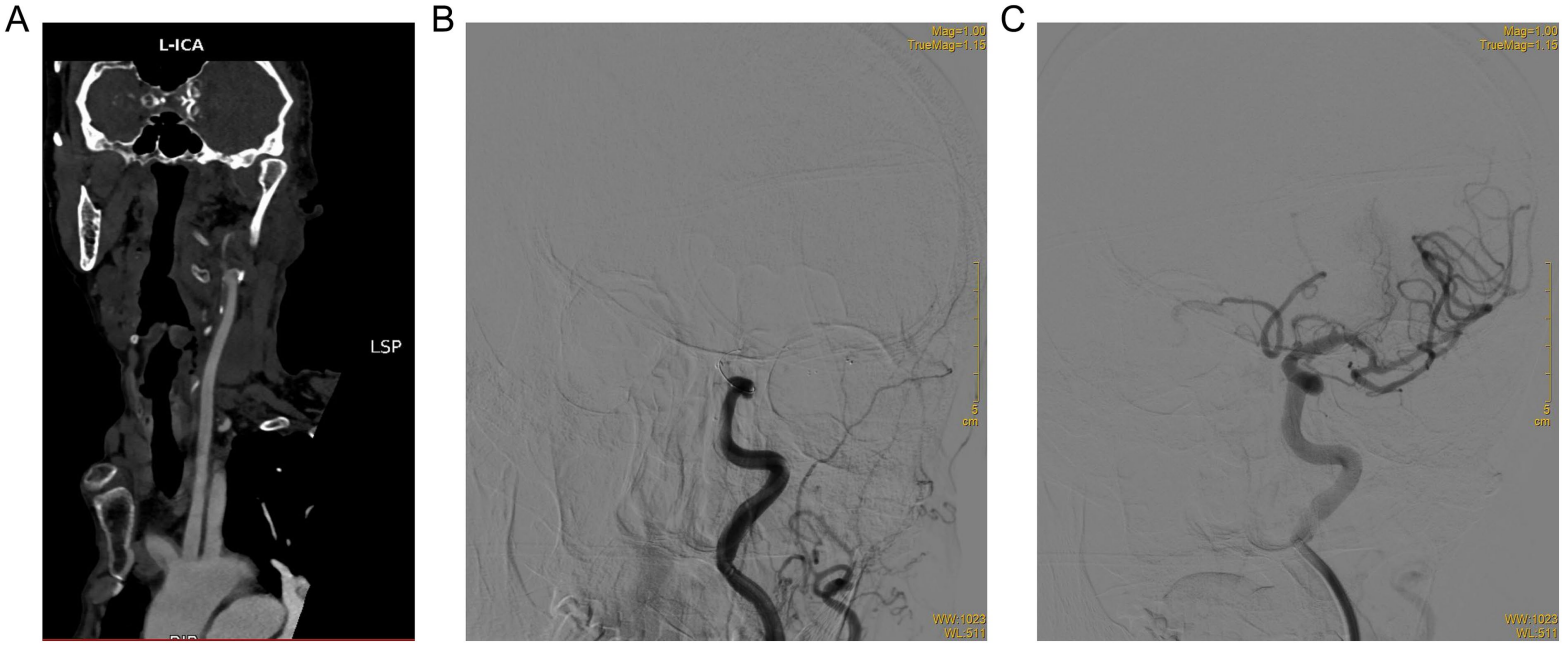
Typical imaging findings of endovascular therapy (EVT) in a patient with acute ischemic stroke due to left internal carotid artery occlusion (0–6 h EVT group). **(A)** Pre-procedural CTA showed complete occlusion of the left internal carotid artery. **(B)** Pre-thrombectomy angiography confirmed the occlusion of the left internal carotid artery. **(C)** Post-thrombectomy angiography achieved successful reperfusion (mTICI 3 grade).

Angiographic and procedural details are summarized in Table 2 and Figure 2. Earlier EVT was associated with both higher-quality and more efficient reperfusion. The proportion of patients achieving successful angiographic reperfusion (mTICI 2b–3) declined from 83.8% in the early group to 76.4% in the intermediate group and 68.6% in the late group (*p* = 0.047). A similar gradient was observed for first-pass effect (50.0, 37.5, and 30.0%; *p* = 0.045), as illustrated in Figure 2A, where later treatment windows are associated with a progressive loss of optimal reperfusion. Typical imaging findings of successful EVT for anterior circulation LVO (left internal carotid artery occlusion) in the early EVT group are presented in Figure 3, which demonstrated the complete process from vascular occlusion to successful reperfusion after thrombectomy.

**Table 2:** Procedural performance and angiographic reperfusion outcomes by EVT timing.

Variable	Early EVT (0–6 h, *n* = 74)	Intermediate EVT (6–12 h, *n* = 72)	Late EVT (12–24 h, *n* = 70)	Statistic	*p* value
Angiographic efficacy					
Successful reperfusion (mTICI 2b–3)	62 (83.8%)	55 (76.4%)	48 (68.6%)	*χ*^2^ = 4.615	0.047
First-pass effect	37 (50.0%)	27 (37.5%)	21 (30.0%)	*χ*^2^ = 6.184	0.045
Number of device passes	1.63 ± 0.71	1.97 ± 0.82	2.21 ± 0.89	*F* = 11.302	<0.001
Procedural strategy					
ADAPT-first	44 (59.5%)	42 (58.3%)	40 (57.1%)	*χ*^2^ = 0.079	0.961
Stent retriever–first	19 (25.7%)	20 (27.8%)	19 (27.1%)	*χ*^2^ = 0.087	0.958
Combined thrombectomy (aspiration + stent retriever)	11 (14.9%)	10 (13.9%)	11 (15.7%)	*χ*^2^ = 0.094	0.954
Rescue therapy (angioplasty or permanent stenting)	8 (10.8%)	9 (12.5%)	13 (18.6%)	χ^2^ = 1.986	0.371
Procedural safety/periprocedural events					
Distal procedural embolization (DPE)	4 (5.4%)	6 (8.3%)	9 (12.9%)	*χ*^2^ = 2.519	0.284
Intra-arterial antithrombotics	5 (6.8%)	8 (11.1%)	11 (15.7%)	*χ*^2^ = 2.922	0.232
Vessel injury	1 (1.4%)	2 (2.8%)	3 (4.3%)	*χ*^2^ = 1.147	0.564
Procedure time, min	46.2 ± 18.1	54.8 ± 20.9	62.1 ± 22.6	*F* = 14.600	<0.001

Continuous variables are presented as mean ± SD and categorical variables as %. Successful reperfusion was defined as mTICI 2b–3. First-pass effect corresponded to mTICI 2b–3 following a single thrombectomy attempt. Rescue therapy included balloon angioplasty or permanent stenting. EVT, endovascular therapy; mTICI, modified Thrombolysis in Cerebral Infarction; ADAPT, direct aspiration first-pass technique; IA, intra-arterial; DPE, distal procedural embolization.

Procedural efficiency also deteriorated with increasing onset-to-groin time (Figure 2B). The mean number of thrombectomy passes increased from 1.63 ± 0.71 to 1.97 ± 0.82 and 2.21 ± 0.89 across the early, intermediate, and late groups (*p* < 0.001), and procedure duration lengthened from 46.2 ± 18.1 to 54.8 ± 20.9 and 62.1 ± 22.6 min (*p* < 0.001). In contrast, the distribution of first-line thrombectomy strategy (aspiration-first, stent retriever–first, or combined), the use of rescue angioplasty or stenting, and periprocedural intra-arterial medications did not differ significantly among groups (all *p* > 0.05), indicating that operator technique and device selection were largely consistent irrespective of treatment timing (Table 2).

### Temporal evolution of neuroinflammatory biomarkers

**Figure 4:**
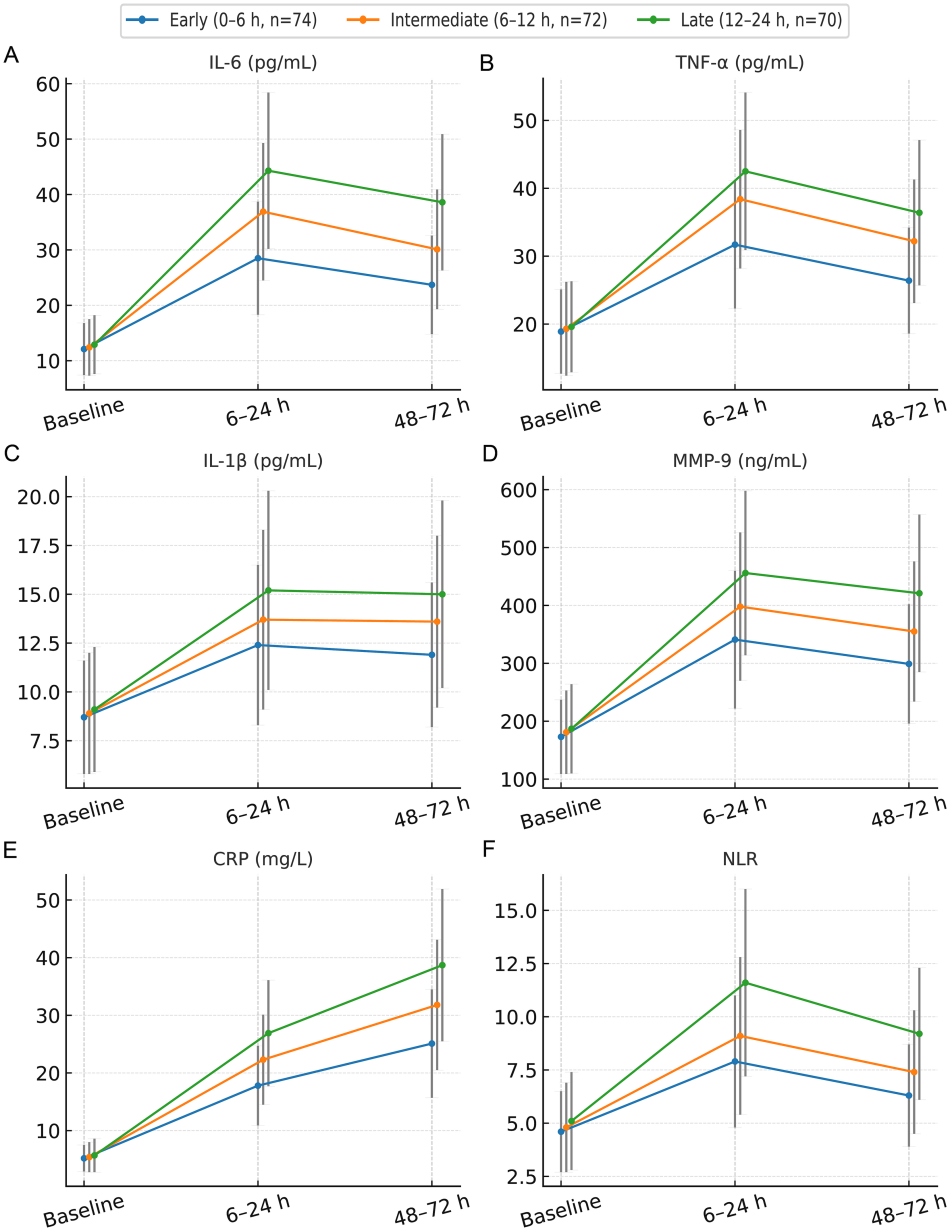
Temporal evolution of circulating neuroinflammatory biomarkers according to endovascular therapy timing. Serial changes in circulating neuroinflammatory biomarkers are shown at baseline, 6–24 h, and 48–72 h after symptom onset in patients undergoing early (0–6 h, *n* = 74), intermediate (6–12 h, *n* = 72), and late (12–24 h, *n* = 70) endovascular therapy (EVT). **(A)** IL-6, **(B)** TNF-*α*, **(C)** IL-1*β*, **(D)** MMP-9, **(E)** C-reactive protein (CRP), and **(F)** neutrophil-to-lymphocyte ratio (NLR). Each panel displays mean values with standard deviations (error bars) for the three EVT timing groups. Overall temporal trends and between-group differences were evaluated using repeated-measures/mixed-effects models.

Serial neuroinflammatory biomarker levels at baseline, 6–24 h, and 48–72 h are summarized in Table 3 and visualized in Figure 4. Baseline concentrations of IL-6, TNF-*α*, IL-1β, MMP-9, CRP, and NLR were comparable across EVT timing strata (all *p* > 0.05). After EVT, earlier treatment was associated with a consistently attenuated systemic inflammatory response and a more rapid normalization of IL-6, TNF-α, MMP-9, CRP, and NLR over follow-up (all *p* < 0.001), whereas IL-1β showed weaker timing sensitivity. Mixed-effects models confirmed significant time-by-timing-group interactions for IL-6, MMP-9, CRP, and NLR (Table 4), supporting that EVT timing was associated with differences in biomarker trajectories rather than isolated single-timepoint shifts.

**Table 3:** Temporal profiles of circulating neuroinflammatory biomarkers following EVT.

Biomarker	Timepoint	Early EVT (0–6 h, *n* = 74)	Intermediate EVT (6–12 h, *n* = 72)	Late EVT (12–24 h, *n* = 70)	*F* statistic	*p* value
IL-6, pg./mL	Baseline	12.1 ± 4.7	12.4 ± 5.1	12.9 ± 5.3	0.391	0.678
	6–24 h	28.5 ± 10.2	36.9 ± 12.4	44.3 ± 14.1	22.426	<0.001
	48–72 h	23.7 ± 8.9	30.1 ± 10.8	38.6 ± 12.3	19.115	<0.001
TNF-α, pg./mL	Baseline	18.9 ± 6.2	19.3 ± 6.9	19.6 ± 6.7	0.294	0.748
	6–24 h	31.7 ± 9.4	38.4 ± 10.2	42.5 ± 11.6	14.893	<0.001
	48–72 h	26.4 ± 7.8	32.2 ± 9.1	36.4 ± 10.7	12.515	<0.001
IL-1β, pg./mL	Baseline	8.7 ± 2.9	8.9 ± 3.1	9.1 ± 3.2	0.523	0.595
	6–24 h	12.4 ± 4.1	13.7 ± 4.6	15.2 ± 5.1	6.192	0.002
	48–72 h	11.9 ± 3.7	13.6 ± 4.4	15.0 ± 4.8	7.131	0.001
MMP-9, ng/mL	Baseline	173 ± 64	181 ± 72	187 ± 77	0.810	0.446
	6–24 h	341 ± 119	398 ± 128	456 ± 142	18.611	<0.001
	48–72 h	299 ± 103	355 ± 121	421 ± 136	11.427	<0.001
CRP, mg/L	Baseline	5.2 ± 2.3	5.4 ± 2.6	5.7 ± 2.9	0.486	0.619
	6–24 h	17.8 ± 6.9	22.3 ± 7.8	26.9 ± 9.2	15.702	<0.001
	48–72 h	25.1 ± 9.4	31.8 ± 11.3	38.7 ± 13.2	18.914	<0.001
NLR	Baseline	4.6 ± 1.9	4.8 ± 2.1	5.1 ± 2.3	1.021	0.361
	6–24 h	7.9 ± 3.1	9.1 ± 3.7	11.6 ± 4.4	19.202	<0.001
	48–72 h	6.3 ± 2.4	7.4 ± 2.9	9.2 ± 3.1	15.113	<0.001

Serial plasma levels of IL-6, TNF-α, IL-1β, MMP-9, CRP, and NLR at baseline, 6–24 h, and 48–72 h after EVT are presented as mean ± SD. IL-6, interleukin-6; TNF-α, tumor necrosis factor-α; IL-1β, interleukin-1β; MMP-9, matrix metalloproteinase-9; CRP, C-reactive protein; NLR, neutrophil-to-lymphocyte ratio.

**Table 4:** Linear mixed-effects models of biomarker trajectories by EVT timing.

Biomarker	Fixed effect (Time × Early EVT)	β (SE)	95% CI	*t* statistic	*p* value
IL-6 (pg/mL)	Time × Early EVT (0–6 h)	−8.21 (2.27)	−12.7 to −3.7	−3.612	< 0.001
TNF-α (pg/mL)	Time × Early EVT (0–6 h)	−1.92 (0.98)	−3.6 to −0.2	−1.963	0.049
IL-1β (pg/mL)	Time × Early EVT (0–6 h)	−0.88 (0.57)	−2.0 to 0.3	−1.531	0.127
MMP-9 (ng/mL)	Time × Early EVT (0–6 h)	−16.40 (4.90)	−25.9 to −7.0	−3.330	0.001
CRP (mg/L)	Time × Early EVT (0–6 h)	−6.70 (2.90)	−12.5 to −0.9	−2.316	0.022
NLR	Time × Early EVT (0–6 h)	−1.06 (0.42)	−1.9 to −0.2	−2.524	0.013

Fixed-effect coefficients are derived from linear mixed-effects models evaluating the interaction between time (repeated measures) and early EVT (0–6 h) compared with later EVT (≥6 h). Values are presented as β (SE), 95% confidence interval, t statistic, and P value. EVT, endovascular therapy; IL-6, interleukin-6; TNF-α, tumor necrosis factor-α; IL-1β, interleukin-1β; MMP-9, matrix metalloproteinase-9; CRP, C-reactive protein; NLR, neutrophil-to-lymphocyte ratio; β, regression coefficient; SE, standard error; CI, confidence interval.

### Radiographic ischemic injury and hemorrhagic transformation

**Figure 5:**
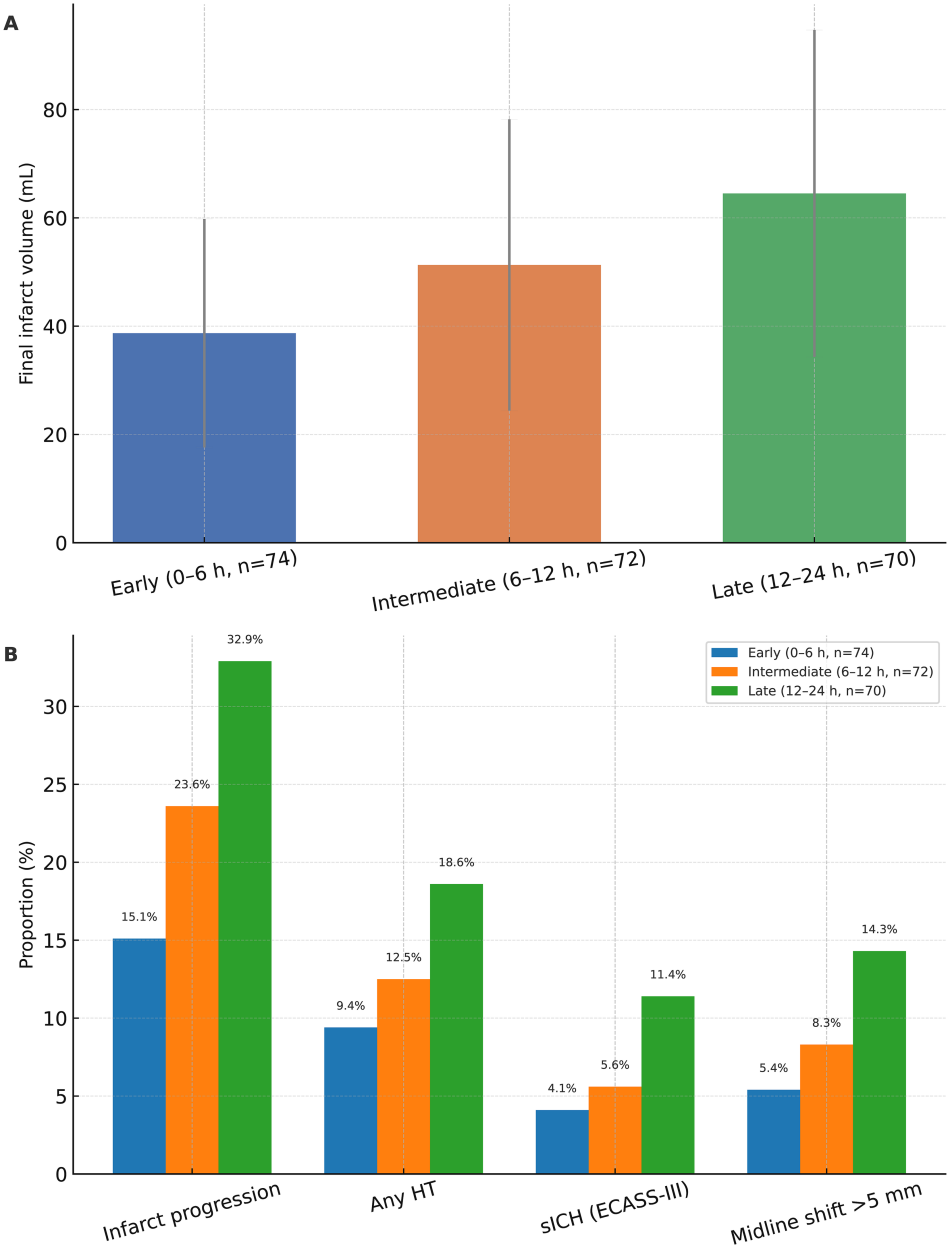
Radiographic outcomes according to endovascular therapy timing. **(A)** Final infarct volume on follow-up imaging in patients undergoing early (0–6 h, *n* = 74), intermediate (6–12 h, *n* = 72), and late (12–24 h, *n* = 70) endovascular therapy (EVT). Bars represent mean values and error bars indicate standard deviation. **(B)** Proportions of patients with infarct progression, any hemorrhagic transformation (HT), symptomatic intracranial hemorrhage (sICH) according to ECASS-III criteria, and midline shift >5 mm across the three EVT timing groups. Values are shown as percentages above each bar. Between-group comparisons for continuous and categorical variables were performed using one-way analysis of variance and χ^2^ tests, respectively.

Radiographic outcomes are summarized in Table 5 and depicted in Figure 5. Final infarct volume at 48–72 h increased stepwise with treatment delay (*p* < 0.001; Figure 5A), with mean volumes of 38.7 ± 11.1 mL in the early group, 51.3 ± 16.9 mL in the intermediate group, and 64.5 ± 20.2 mL in the late group. This gradient indicates a substantially lower infarct burden among patients treated within 0–6 h compared with those treated later. Consistent with the volumetric findings, categorical imaging endpoints—including infarct progression, hemorrhagic transformation (including sICH), and midline shift—showed a directionally less favorable profile with increasing delay, as detailed in Table 5 and visualized in Figure 5B.

**Table 5:** Radiographic outcomes including infarct burden, hemorrhagic complications, and mass effect across EVT timing groups.

Radiographic parameter	Early EVT (0–6 h)	Intermediate EVT (6–12 h)	Late EVT (12–24 h)	Statistic	*p* value
Final infarct volume, mL	38.7 ± 11.1	51.3 ± 16.9	64.5 ± 20.2	*F* = 12.817	< 0.001
Infarct progression	14/93 (15.1%)	20/85 (23.6%)	26/79 (32.9%)	*χ*^2^ = 7.614	0.022
Any hemorrhagic transformation (HT)	9/96 (9.4%)	11/88 (12.5%)	17/91 (18.6%)	*χ*^2^ = 3.576	0.167
sICH (ECASS-III)	4/97 (4.1%)	5/90 (5.6%)	10/88 (11.4%)	*χ*^2^ = 4.141	0.126
Midline shift >5 mm	4/74 (5.4%)	6/72 (8.3%)	10/70 (14.3%)	*χ*^2^ = 3.487	0.175

Radiographic outcomes were assessed using follow-up neuroimaging. Final infarct volume was quantified on 48–72 h diffusion-weighted MRI or noncontrast CT. Infarct progression was defined as a ≥ 30% increase in infarct burden relative to baseline ASPECTS-adjusted infarct core on 48–72 h imaging. Hemorrhagic complications include any hemorrhagic transformation and symptomatic intracerebral hemorrhage according to ECASS-III criteria. EVT, endovascular therapy; HT, hemorrhagic transformation; sICH, symptomatic intracerebral hemorrhage; ASPECTS, Alberta Stroke Program Early CT Score; MRI, magnetic resonance imaging; CT, computed tomography.

### Neurological recovery and 90-day clinical outcomes

**Figure 6:**
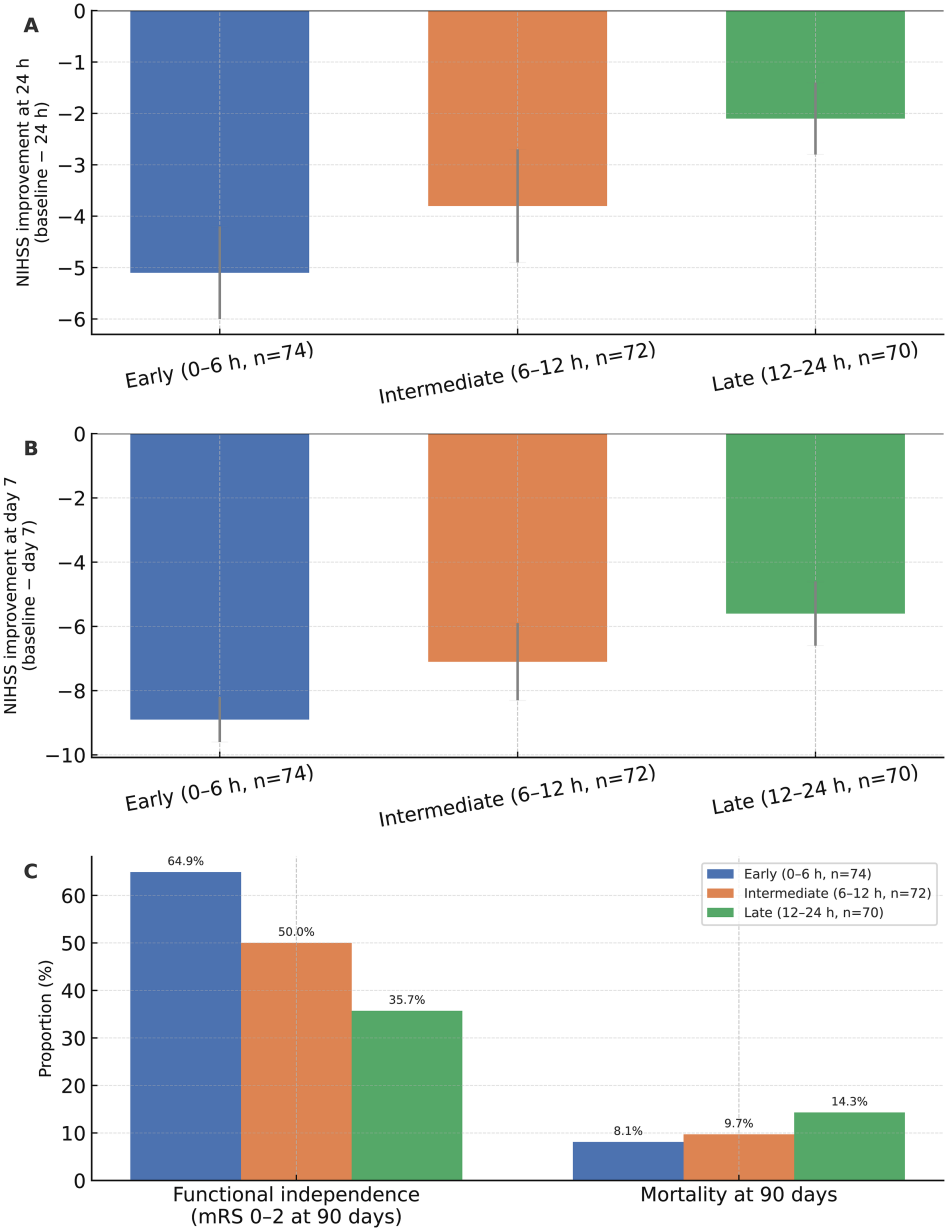
Functional and clinical outcomes according to endovascular therapy timing. **(A)** National Institutes of Health Stroke Scale (NIHSS) improvement at 24 h, expressed as the change from baseline to 24 h (baseline − 24 h), in patients undergoing early (0–6 h, *n* = 74), intermediate (6–12 h, *n* = 72), and late (12–24 h, *n* = 70) endovascular therapy (EVT). Bars represent mean values and error bars indicate standard deviation. **(B)** NIHSS improvement at day 7 (baseline − day 7) across the three EVT timing groups, presented as mean ± standard deviation. Negative values indicate greater neurological improvement. **(C)** Proportions of patients achieving functional independence [modified Rankin Scale (mRS) score 0–2 at 90 days] and 90-day mortality in each EVT timing group. Values are shown as percentages above each bar. Between-group differences for continuous and categorical outcomes were assessed using one-way analysis of variance and χ^2^ tests, respectively.

Neurological improvement and long-term clinical outcomes according to EVT timing are detailed in Table 6 and Figure 6. Early EVT was associated with more pronounced early neurological recovery. At 24 h, mean NIHSS change (baseline − 24 h) was −5.1 ± 0.9 in the early group, compared with −3.8 ± 1.1 and −2.1 ± 0.7 in the intermediate and late groups, respectively (*p* < 0.001). By day 7, this difference persisted (−8.9 ± 0.7 *vs* −7.1 ± 1.2 *vs* −5.6 ± 1.0; *p* = 0.001), indicating sustained benefits of earlier intervention (Figures 6A,B).

**Table 6:** Early neurological recovery and long-term functional independence by EVT timing.

Outcome	Early EVT (0–6 h, *n* = 74)	Intermediate EVT (6–12 h, *n* = 72)	Late EVT (12–24 h, *n* = 70)	Statistic	*p* value
NIHSS improvement at 24 h (baseline − 24 h)	−5.1 ± 0.9	−3.8 ± 1.1	−2.1 ± 0.7	*F* = 13.423	< 0.001
NIHSS improvement at day 7	−8.9 ± 0.7	−7.1 ± 1.2	−5.6 ± 1.0	*F* = 7.992	0.001
mRS 0–2 at 90 days	48 (64.9%)	36 (50.0%)	25 (35.7%)	*χ*^2^ = 12.237	0.002
Ordinal mRS shift (0–6 vs. 6–12 vs. 12–24 h)	—	—	—	LR *χ*^2^ = 21.752	< 0.001
Mortality at 90 days	6 (8.1%)	7 (9.7%)	10 (14.3%)	*χ*^2^ = 1.540	0.463

ΔNIHSS was calculated as baseline score minus follow-up score, such that negative values indicate neurological improvement. Ordinal mRS shift was assessed using proportional odds modeling across full modified Rankin Scale categories. Mortality was recorded as all-cause death at 90 days. EVT, endovascular therapy; NIHSS, National Institutes of Health Stroke Scale; mRS, modified Rankin Scale; LR *χ*^2^, likelihood ratio chi-square.

These early gains were accompanied by superior 90-day functional outcomes. Follow-up ascertainment for the primary endpoint was complete, with 90-day mRS available for all patients (216/216). The proportion of patients achieving functional independence (mRS 0–2 at 90 days) was highest in the early EVT group (48/74, 64.9%), intermediate in the 6–12 h group (36/72, 50.0%), and lowest in the 12–24 h group (25/70, 35.7%; *p* = 0.002). Ordinal shift analysis across the full mRS distribution demonstrated a significant global improvement in disability profile with earlier treatment (likelihood ratio *χ^2^* = 21.752; *p* < 0.001), reinforcing the graded relationship between EVT timing and functional recovery. In contrast, 90-day all-cause mortality did not differ significantly across groups (8.1% *vs* 9.7% *vs* 14.3%; *p* = 0.463), although absolute mortality was lowest in the early EVT cohort (Figure 6C). In prespecified adjusted analyses, the association between earlier EVT and better functional outcome remained robust. In multivariable logistic regression adjusting for age, baseline NIHSS, baseline ASPECTS, occlusion segment, IV thrombolysis, and successful reperfusion, patients treated within 0–6 h had higher odds of functional independence at 90 days compared with those treated at 12–24 h (adjusted OR, 3.32; 95% CI, 1.68–6.58; *p* < 0.001), with consistent estimates when compared with the 6–12 h group (adjusted OR, 1.80; 95% CI, 0.92–3.53; *p* = 0.093). This association remained robust after additional adjustment for baseline infarct core volume (adjusted OR = 3.15, 95% CI = 1.56–6.36, *p* < 0.001). There was no evidence of effect modification by anesthesia modality for the association between EVT timing and 90-day functional independence (*P*_interaction = 0.442). In an adjusted proportional-odds model, earlier EVT was also associated with a favorable shift across the full mRS distribution (common OR for better outcome, 1.22; 95% CI, 1.04–1.41; *p* < 0.001). Findings were concordant in stabilized inverse probability–weighted models (weighted OR for mRS 0–2, 1.18; 95% CI, 1.04–1.33; *p* < 0.001). Moreover, results were materially unchanged in sensitivity analyses that more explicitly accounted for baseline ASPECTS, including alternative ASPECTS parameterizations (continuous *vs* categorical) and restriction to patients with ASPECTS ≥6 and ≥7, supporting that the observed timing–outcome association was not driven solely by baseline ischemic burden.

### Procedural complications and in-hospital safety

Procedural and in-hospital safety outcomes are summarized in Table 7. The overall incidence of any procedural complication was low and comparable across early, intermediate, and late EVT groups (9.5, 11.1, and 14.3%, respectively; *p* = 0.657). Rates of peri-procedural sICH (4.1% *vs* 5.6% *vs* 11.4%; *p* = 0.187), vessel perforation (0, 1.4, and 2.9%; *p* = 0.342), distal embolization (5.4, 8.3, and 12.9%; *p* = 0.284), and in-hospital mortality (5.4, 6.9, and 10.0%; *p* = 0.565) did not differ significantly among timing strata. These data indicate that accelerating EVT within the first 6 h did not incur an excess procedural or in-hospital safety penalty compared with later intervention.

**Table 7:** Procedural and in-hospital safety outcomes following EVT.

Safety endpoint	Early EVT (0–6 h)	Intermediate EVT (6–12 h)	Late EVT (12–24 h)	Statistic	*p* value
Any procedural complication	7/74 (9.5%)	8/72 (11.1%)	10/70 (14.3%)	*χ*^2^ = 0.841	0.657
Peri-procedural sICH (ECASS-III)	3/74 (4.1%)	4/72 (5.6%)	8/70 (11.4%)	*χ*^2^ = 3.350	0.187
Vessel perforation	0/74 (0%)	1/72 (1.4%)	2/70 (2.9%)	*χ*^2^ = 2.144	0.342
Distal embolization	4/74 (5.4%)	6/72 (8.3%)	9/70 (12.9%)	*χ*^2^ = 2.519	0.284
In-hospital mortality	4/74 (5.4%)	5/72 (6.9%)	7/70 (10.0%)	*χ*^2^ = 1.141	0.565

Procedural complications were adjudicated intraoperatively or during the index hospitalization. Peri-procedural symptomatic intracerebral hemorrhage (sICH) was defined according to ECASS-III criteria. Distal embolization was defined as embolic occlusion of a previously unaffected distal arterial branch during thrombectomy. Vessel perforation included angiographically evident rupture or extravasation requiring bailout measures. EVT, endovascular therapy; sICH, symptomatic intracerebral hemorrhage; ECASS-III, European Cooperative Acute Stroke Study III.

### Covariate balance in weighted analyses

**Figure 7:**
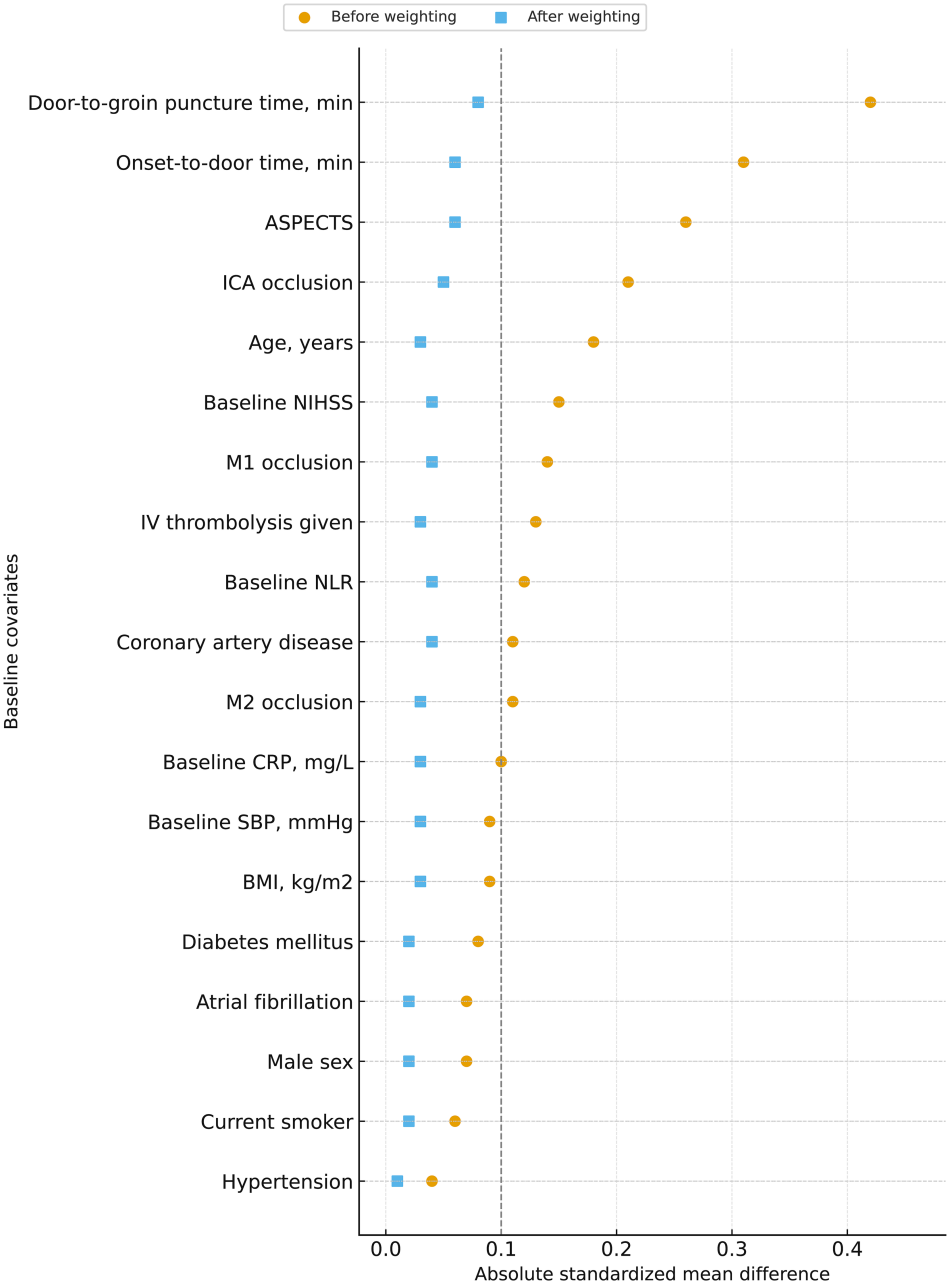
Covariate balance before and after weighting between early and late EVT groups. Absolute standardized mean differences (SMDs) for baseline clinical, imaging, workflow, and inflammatory covariates are shown before and after application of the weighting procedure. Circles indicate the absolute SMD for each covariate when comparing early and delayed endovascular therapy (EVT) groups before weighting, and squares indicate the corresponding SMDs after weighting. The vertical dashed line at an absolute SMD of 0.10 denotes a conventional threshold for acceptable imbalance. After weighting, all covariates shifted substantially toward this threshold, indicating improved baseline balance between groups.

Inverse probability weighting was applied to mitigate treatment-selection bias in exploratory analyses comparing early and late EVT. Before weighting, several baseline variables—including ASPECTS and key workflow times—showed moderate imbalance between groups. After weighting, standardized mean differences for all covariates were reduced to ≤0.08, with most falling below the prespecified threshold of 0.10, indicating satisfactory post-weighting balance (Table 8 and Figure 7). No extreme stabilized weights were observed, supporting the robustness of the weighted models.

**Table 8:** Balance of baseline covariates before and after inverse probability weighting.

Variable	Domain	SMD before weighting	SMD after weighting
Age, years	Demographics	0.18	0.03
Male sex	Demographics	0.07	0.02
BMI, kg/m^2^	Demographics	0.09	0.03
Hypertension	Vascular risk	0.04	0.01
Diabetes mellitus	Vascular risk	0.08	0.02
Atrial fibrillation	Vascular risk	0.07	0.02
Coronary artery disease	Vascular risk	0.11	0.04
Current smoker	Vascular risk	0.06	0.02
Baseline NIHSS	Stroke severity	0.15	0.04
ASPECTS	Stroke severity	0.26	0.06
Baseline SBP, mmHg	Stroke severity	0.09	0.03
ICA occlusion	Occlusion site	0.21	0.05
M1 occlusion	Occlusion site	0.14	0.04
M2 occlusion	Occlusion site	0.11	0.03
IV thrombolysis given	Acute treatment	0.13	0.03
Onset-to-door time, min	Workflow time	0.31	0.06
Door-to-groin puncture time, min	Workflow time	0.42	0.08
Baseline CRP, mg/L	Inflammatory profile	0.10	0.03
Baseline NLR	Inflammatory profile	0.12	0.04

Standardized mean differences (SMDs) are reported before and after inverse probability weighting (IPW). IPW was applied to balance baseline covariates across EVT timing groups using a treatment assignment model. SMD <0.10 was considered indicative of adequate balance. No extreme stabilized weights (>10 or <0.1) were observed. IPW, inverse probability weighting; NIHSS, National Institutes of Health Stroke Scale; ASPECTS, Alberta Stroke Program Early CT Score; CRP, C-reactive protein; NLR, neutrophil-to-lymphocyte ratio; SBP, systolic blood pressure; EVT, endovascular therapy.

## Discussion

In this cohort of anterior-circulation LVO treated within 24 h, earlier EVT was associated with superior recovery, including smaller infarct volumes, attenuated systemic inflammation, and better functional outcomes. These findings reinforce the importance of EVT timing in influencing both immediate injury and downstream recovery mechanisms. A key contribution of this study is the integration of serial inflammatory profiling with EVT timing. The observed attenuation and faster resolution of systemic inflammatory markers with earlier reperfusion is biologically plausible given experimental evidence linking delayed reperfusion to microvascular dysfunction, blood–brain barrier disruption, and amplification of innate immune signaling (32–37). Importantly, these analytes were measured in peripheral blood and should be interpreted as systemic correlates consistent with differential inflammatory activation, rather than direct evidence of specific CNS-compartment cellular mechanisms (38–45).

From a clinical perspective, the biomarker gradients appear potentially meaningful in magnitude rather than merely statistically detectable and may help motivate future work on risk stratification and adjunctive therapies targeting inflammation or barrier stability alongside EVT. Nevertheless, circulating measures are not disease-specific and were not used to guide management in this cohort; therefore, they should be viewed as clinically relevant correlates rather than definitive surrogates of brain-compartment inflammation. Beyond mechanistic insight, our findings highlight the importance of continued efforts to shorten onset-to-reperfusion intervals. Imaging-based selection has expanded treatment opportunities, but does not negate the progressive biological costs of delay. Workflow optimization, prehospital triage, and real-time performance feedback could mitigate neuroinflammation and improve outcomes in patients treated late.

These mechanistic observations align closely with our radiographic and clinical findings. Final infarct volume and radiographic infarct progression rose in a stepwise fashion from the early to the late EVT group, while symptomatic intracranial hemorrhage and midline shift showed only modest, non-significant trends toward increase with treatment delay. This combination—marked reduction in infarct growth without a clear penalty in hemorrhagic complications—is consistent with the hypothesis that early reperfusion limits both direct ischemic necrosis and inflammation-mediated secondary injury. In parallel, patients treated within 0–6 h exhibited greater NIHSS improvement at 24 h and day 7, and a significantly higher probability of achieving functional independence at 90 days, accompanied by a favorable shift in the overall modified Rankin Scale distribution. These data suggest that the clinical benefits of early EVT are not solely a function of restoring large-vessel patency but are also mediated by more subtle biological effects on tissue viability, microcirculatory integrity, and the inflammatory milieu. By demonstrating a coherent chain from EVT timing to reperfusion performance, inflammatory trajectories, infarct burden, and functional outcome, this study helps bridge the gap between bench and bedside concepts of reperfusion–inflammation interactions in acute stroke.

Beyond mechanistic insight, our findings have several practical implications for stroke systems of care and for the design of future EVT studies. First, the data reinforce the notion that time remains critical even within an extended 24-h eligibility window. Imaging-based selection has rightly expanded treatment opportunities for patients presenting late, but it does not negate the progressive biological costs of delay (46). The demonstration that early EVT is associated with a more favorable inflammatory profile and superior clinical outcomes, without an accompanying increase in procedural risk, underscores the importance of continued efforts to shorten onset-to-door, door-to-imaging, and door-to-groin intervals. Workflow optimization, prehospital triage protocols, direct-to-angio pathways, and real-time performance feedback may all yield benefits that extend beyond simple core–penumbra dynamics by also attenuating post-ischemic neuroinflammation. Second, serial biomarker profiling in this cohort highlights the potential value of inflammatory markers as complementary tools for risk stratification and therapeutic monitoring. In the future, inflammatory signatures—integrated with advanced imaging markers of collateral status, tissue perfusion, or microglial activation—could help identify subgroups who might benefit most from ultra-early EVT, adjunctive anti-inflammatory therapies, or intensified post-procedural monitoring.

Several limitations merit consideration. This study was conducted at a single high-volume tertiary center with established EVT workflows and experienced operators, which may limit generalizability to smaller or resource-constrained settings. External validity may also vary across healthcare systems with different prehospital stroke pathways and transfer architectures. In particular, regions that rely predominantly on inter-hospital ‘drip-and-ship’ transfer, have limited 24/7 advanced imaging availability, or use different EMS triage/bypass protocols (e.g., preferential routing of suspected LVO directly to comprehensive stroke centers) may exhibit different distributions of onset-to-door and door-to-groin intervals than our setting. Because EVT timing strata in this study were shaped by real-world recognition, transport, and workflow factors, the magnitude of timing-related gradients in reperfusion efficiency, biomarker trajectories, and outcomes may differ where prehospital screening tools, transport distances, and hub-and-spoke organization are materially different. Although procedures were performed under standardized protocols and the majority of cases were handled by a small group of senior interventionalists, we cannot fully exclude operator-dependent effects or secular improvements over the study period (e.g., evolving device iterations, team coordination, and thresholds for switching or rescue strategies). Such learning-curve and time-trend factors could have modestly influenced procedural efficiency, reperfusion quality, and downstream outcomes independent of EVT timing per se. EVT timing was not randomized; instead, it reflected real-world factors including onset recognition, prehospital delay, inter-hospital transfer pathways, and imaging availability. Although we applied prespecified multivariable adjustment and inverse probability weighting to address measured baseline imbalances, residual confounding by unmeasured factors cannot be fully excluded. In particular, collateral circulation was not systematically graded using a standardized scale across the cohort, and collateral status is closely linked to infarct growth kinetics, tissue viability, and functional outcome. Differential collateral profiles across timing strata could influence both eligibility for late EVT and downstream outcomes, potentially contributing to observed gradients that are not solely attributable to treatment delay. The sample size, while adequate to detect differences in infarct volume and functional independence, was relatively modest for rare safety events (47), and estimates of symptomatic intracranial hemorrhage, vessel perforation, or other procedural complications remain imprecise. Biomarkers were measured in peripheral blood at three timepoints, which provides only an indirect approximation of CNS inflammation. Peripheral concentrations are influenced by systemic comorbidities and peri-procedural stress responses and may not track brain-compartment processes governed by blood–brain barrier integrity, intracranial cytokine gradients, and cell-specific signaling (e.g., microglia- and astrocyte-driven responses). Moreover, certain mediators may exhibit rapid and compartmentalized kinetics with limited spillover into the circulation. Therefore, our biomarker findings should be interpreted as systemic correlates consistent with differential inflammatory activation across timing strata, rather than definitive evidence that earlier EVT directly modulates specific CNS inflammatory pathways. Future studies incorporating CSF sampling and/or inflammation-sensitive neuroimaging would help validate CNS specificity. In addition, for unwitnessed and wake-up strokes, onset time was necessarily approximated using the last-known-well convention, which may overestimate true onset-to-groin time in some patients. Such nondifferential misclassification could shift a subset of patients toward later timing strata and would be expected to attenuate, rather than exaggerate, timing-related differences. Finally, our biomarker panel was intentionally focused on a small set of clinically accessible cytokines and a protease axis; thus, other potentially important inflammatory pathways were not assessed, including chemokine signaling, damage-associated molecular patterns, complement activation, platelet–leukocyte interactions and neutrophil extracellular traps (NETs), and adaptive immune phenotypes.

Alternative approaches could further interrogate and refine the hypothesis that EVT timing modulates neuroinflammation and downstream outcomes. Multicenter observational cohorts with harmonized biomarker protocols, standardized imaging, and detailed workflow data would provide broader external validity and greater power to examine interactions with stroke etiology, collateral status, and individual risk factor profiles. Pragmatic trials could embed mechanistic substudies, collecting serial blood and imaging biomarkers alongside routine clinical data, to assess how novel workflow interventions or device technologies influence both reperfusion quality and inflammatory responses. Parallel experimental work in animal models could dissect cellular and molecular mechanisms that are difficult to study in humans, such as microglial phenotypic shifts, T-cell infiltration, or specific inflammasome pathways, under varying reperfusion delays. In addition, emerging neuroimaging techniques—such as PET tracers targeting microglial activation or BBB permeability mapping—may allow *in vivo* visualization of neuroinflammation in relation to EVT timing and could help identify thresholds beyond which tissue is no longer salvageable despite recanalization.

Looking ahead, several avenues for future research emerge from this work. Multicenter studies are needed to validate the gradients observed here across different health systems, populations, and organizational models, and to clarify whether similar patterns exist for posterior circulation stroke or for patients presenting beyond 24 h under more permissive imaging criteria. The integration of inflammatory biomarkers into acute stroke pathways could facilitate the development of combined strategies that pair ultra-early EVT with targeted immunomodulatory or neuroprotective therapies, aiming to amplify the benefits of reperfusion while minimizing secondary injury. Adaptive platform trials could test combinations of EVT with agents targeting IL-6, MMP-9, microglial activation, or other key nodes in the post-ischemic inflammatory cascade.

Ultimately, these data support a framework in which earlier EVT is associated with more favorable reperfusion performance, a less pronounced systemic inflammatory response, and better functional recovery. However, because EVT timing was not randomized, residual confounding cannot be excluded, and causal inferences regarding inflammatory mechanisms should be avoided. Future multicenter studies with harmonized workflow, imaging, and biomarker assessment are needed to validate these associations.

## Data Availability

The original contributions presented in the study are included in the article/supplementary material, further inquiries can be directed to the corresponding author.
